# Fast assessment of lipid content in arteries *in vivo* by intravascular photoacoustic tomography

**DOI:** 10.1038/s41598-018-20881-5

**Published:** 2018-02-05

**Authors:** Yingchun Cao, Ayeeshik Kole, Jie Hui, Yi Zhang, Jieying Mai, Mouhamad Alloosh, Michael Sturek, Ji-Xin Cheng

**Affiliations:** 1 Weldon School of Biomedical Engineering, Purdue University, West Lafayette, Indiana 47907 USA; 2 Department of Cellular & Integrative Physiology, Indiana University School of Medicine, Indianapolis, Indiana 46202 USA; 3 Department of Physics and Astronomy, Purdue University, West Lafayette, Indiana 47907 USA; 4 Department of Physics, Boston University, Boston, Massachusetts 02215 USA; 5 Department of Chemistry, Purdue University, West Lafayette, Indiana 47907 USA; 6 Department of Electrical and Computer Engineering, Boston University, Boston, Massachusetts 02215 USA; 7 Department of Biomedical Engineering, Boston University, Boston, Massachusetts 02215 USA

## Abstract

Intravascular photoacoustic tomography is an emerging technology for mapping lipid deposition within an arterial wall for the investigation of the vulnerability of atherosclerotic plaques to rupture. By converting localized laser absorption in lipid-rich biological tissue into ultrasonic waves through thermoelastic expansion, intravascular photoacoustic tomography is uniquely capable of imaging the entire arterial wall with chemical selectivity and depth resolution. However, technical challenges, including an imaging catheter with sufficient sensitivity and depth and a functional sheath material without significant signal attenuation and artifact generation for both photoacoustics and ultrasound, have prevented *in vivo* application of intravascular photoacoustic imaging for clinical translation. Here, we present a highly sensitive quasi-collinear dual-mode photoacoustic/ultrasound catheter with elaborately selected sheath material, and demonstrated the performance of our intravascular photoacoustic tomography system by *in vivo* imaging of lipid distribution in rabbit aortas under clinically relevant conditions at imaging speeds up to 16 frames per second. *Ex vivo* evaluation of fresh human coronary arteries further confirmed the performance of our imaging system for accurate lipid localization and quantification of the entire arterial wall, indicating its clinical significance and translational capability.

## Introduction

Coronary artery disease is the leading cause of mortality worldwide^1^. The disease refers to the pathologic development of atheromatous plaques in the coronary arterial tree and the subsequent narrowing of the lumen or even formation of thrombus due to plaque rupture, leading to restriction of blood flow and life-threatening acute coronary syndrome^2^. Plaques that are considered most susceptible to rupture, or vulnerable plaques, are those with a large lipid-rich necrotic core, covered by a thin fibrous cap, and dense inflammatory infiltrate^3,4^. Reliable and accurate detection of vulnerable plaques would ideally include not only morphological information of the artery wall, but also chemical composition of the suspected lesion^5^. Intravascular ultrasound (IVUS)^6^ and optical coherence tomography^7^ can provide important morphological information of an artery. However, they lack chemical selectivity to accurately assess plaque composition^8,9^. Near-infrared spectroscopy combined with IVUS has been shown to detect the presence of lipid-rich plaques and quantify them with a lipid core burden index^10,11^, yet lack depth resolution to quantify and localize the cholesterol accumulation in lipid-rich plaques.

Intravascular photoacoustic (IVPA) tomography is an emerging catheter-based technology for the localization, quantification, and characterization of lipid deposition while simultaneously complementing traditional IVUS^12^. The biggest advantage is that it can provide lipid-specific detection with depth resolution over the entire arterial wall by converting light absorption into ultrasound (US) detection^13,14^. Over the past several years, efforts have been made towards technical improvement of IVPA technique to meet clinical requirements including the report of various catheter designs^14–18^, the development of laser sources for increased lipid sensitivity and imaging speeds^18–22^, and differentiation of multiple tissue components^23–25^. Nevertheless, catheter sensitivity in current designs has been the biggest obstacle for *in vivo* demonstration. Front-and-back designs exhibit insufficient depth range to encompass the entire artery wall^15–17,26^; co-axial designs are limited by transducer dimensions making the catheter too large for coronary artery access^19^; Co-linear catheter designs have shown improved photoacoustic (PA) sensitivity and depth, but poor US resolution due to considerable signal loss at multiple reflective surfaces^14,22^. In addition, a proper protective sheath material that is transparent to both PA and US signals is essential for *in vivo* application, but has yet to be identified. *In vivo* IVPA imaging was previously attempted in animal models^27–30^, however, incomplete technical preparations, such as lack of a protective sheath^27,28^, lack of morphological feature provided by US^29^, and artificial plaque, blood clearance and unsuitable sheath material^30^, prevent them from functioning well and providing valuable information under clinically relevant conditions.

In this work, we developed a quasi-collinear IVPA catheter with high sensitivity and sufficient depth and selected a sheath material with minimal PA and US attenuation and artifact generation. These advantages enabled *in vivo* IVPA imaging of native arteries in a rabbit model under clinically-relevant conditions with real-time display up to 16 frames per second (fps). We performed localization and quantification of lipid content along the full depth of the arterial wall from intima to perivascular adipose tissue for pullback lengths up to 80 mm.

## Results

### Performance of quasi-collinear IVPA catheter

**Figure 1:**
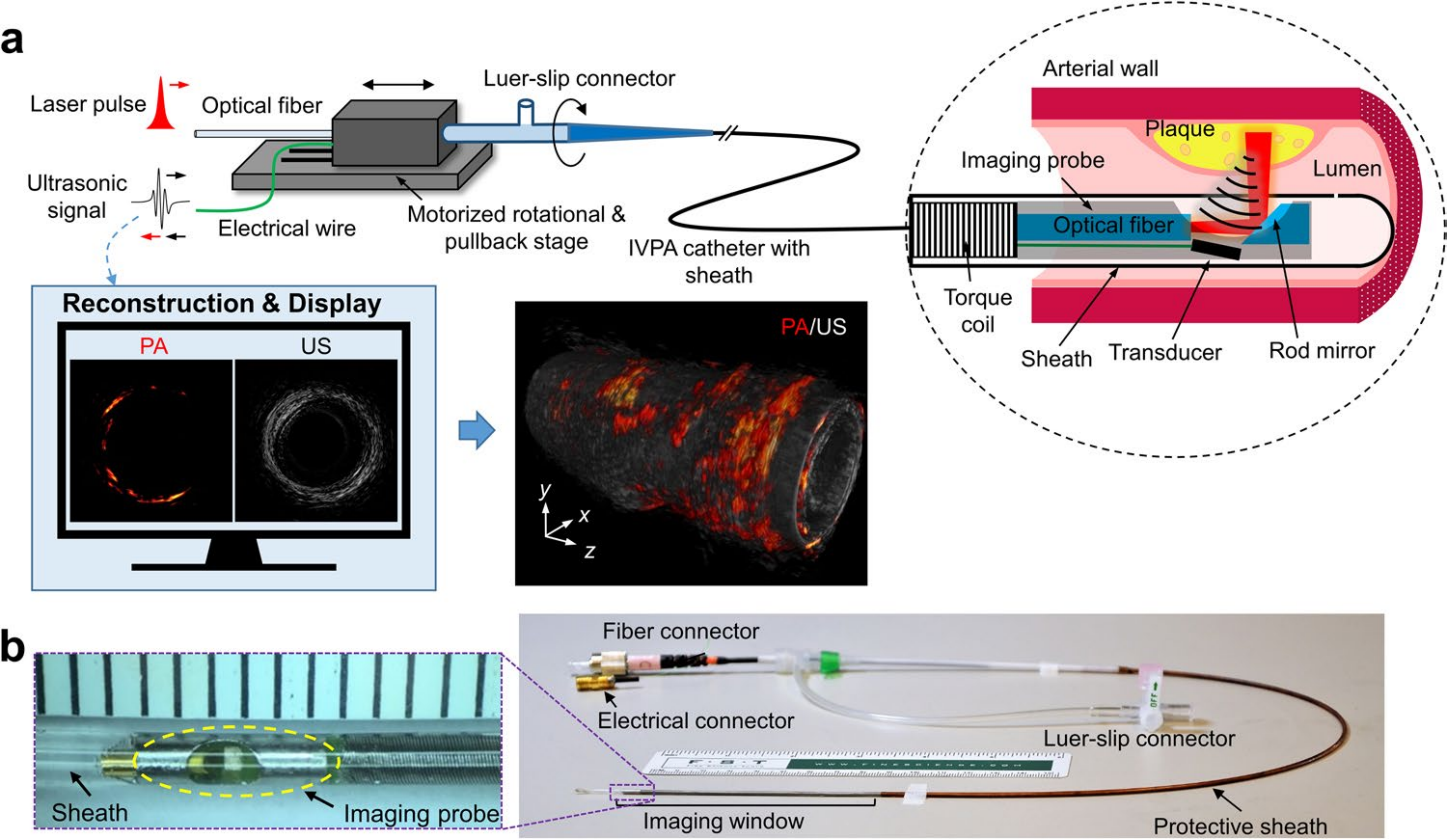
Illustration of IVPA imaging and fabricated catheter. (**a**) Implementation of IVPA imaging. Optical pulses from the excitation laser are coupled to the imaging catheter through a multimode fiber and a rotary joint, and directed to the arterial wall for PA excitation. The generated sound wave from optical absorption by lipid is collected by a transducer installed in the catheter tip. Simultaneously, a delayed ultrasonic pulse is delivered and its echo is received by the same transducer to produce a co-registered US image. The rotational and pullback stages are used to constantly rotate and linearly pull back the IVPA catheter inside a protective sheath for 3D imaging. The reconstructed PA and US images are displayed on a monitor in real time. 3D images of the artery are reconstructed from the cross-sectional image stacks in a pullback. (**b**) Photograph of quasi-collinear IVPA catheter with a complete sheath. The unit scale of the ruler in the left inset of **b** is 1 mm.

**Figure 2:**
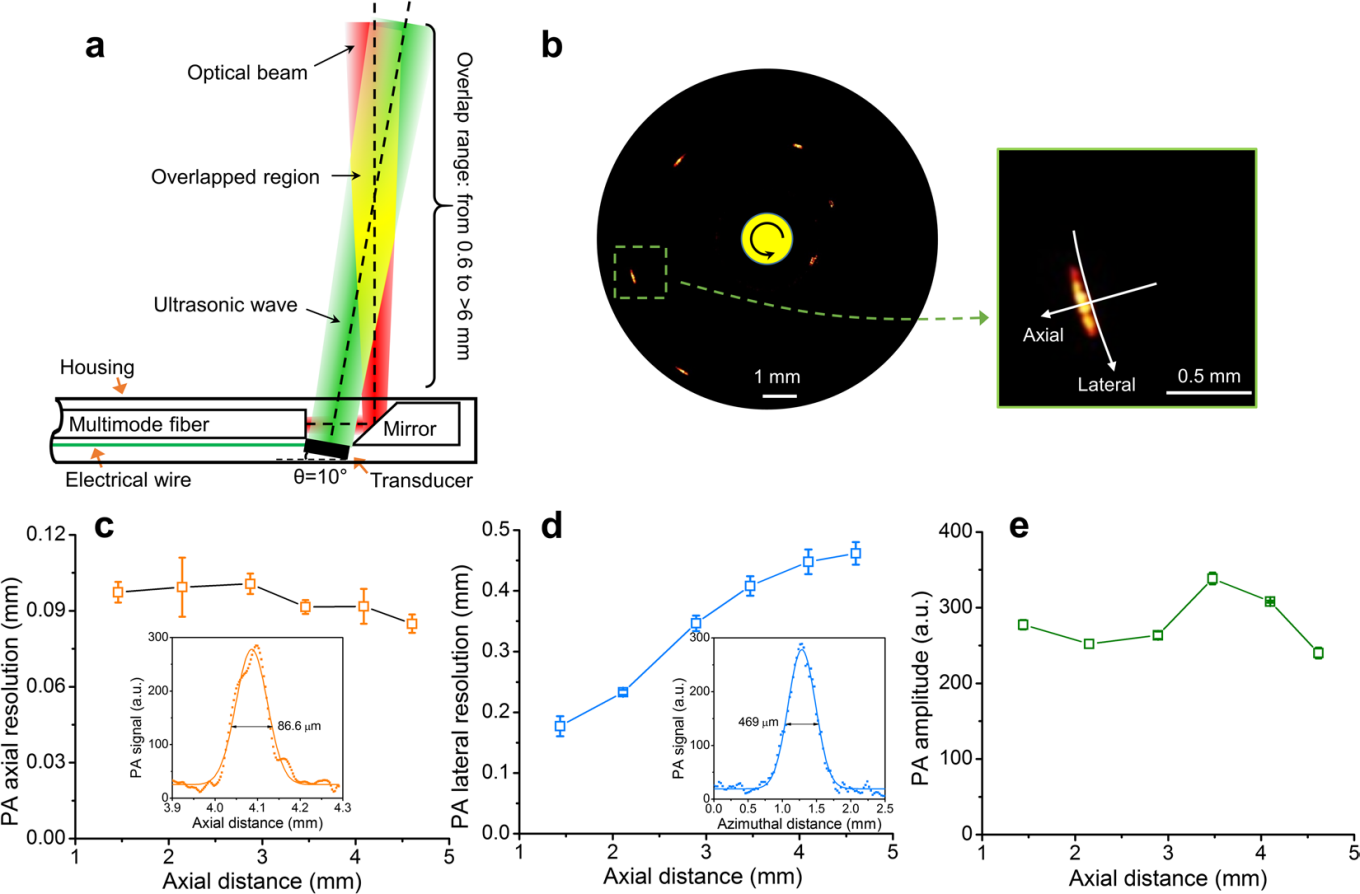
Design and evaluation of a quasi-collinear IVPA catheter. (**a**) Schematic of quasi-collinear IVPA catheter design showing PA imaging depth ranging from 0.6 to >6 mm based on estimated divergence angles of 3° and 6° for ultrasound and optical beams, respectively. (**b**) Combined PA images of a 7-µm carbon fiber at different distances from the catheter center from 1.4 to 4.6 mm. The insets showing the photo of the catheter tip and enlarged image of the target at a distance of 4.1 mm. (**c**) PA axial and (**d**) lateral resolutions, and (**e**) amplitude with insets in c and d showing the PA signals across the target at an axial distance of 4.1 mm along axial and lateral directions, respectively. Error bars in **c**-**e** were generated from three repeated measurements at each location.

IVPA tomography is a hybrid intravascular imaging technology that combines the advantages of optical absorption-based contrast for depth-resolved lipid-specific mapping and traditional US detection for deep tissue morphology (Fig. 1a). Currently, sensitivity remains the most important technical challenge for IVPA to be applied to *in vivo* study. To address this problem, we developed a quasi-collinear catheter (see Methods, Figs 1b and 2a), the diameter of which including outer sheath was measured to be 1.6 mm at the tip (Fig. 1b), and integrated it with our high-speed IVPA imaging system (Supplementary Fig. S1)^22^. The spatial resolution and imaging depth of the catheter with protective sheath was evaluated by imaging a 7-µm carbon fiber placed at different distances from the probe as shown in Fig. 2b. To maintain a detectable PA signal for a small target, the experiments were performed in deuterium oxide (D_2_O) to reduce optical attenuation in the medium. The axial resolutions are measured to range from 85 to 100 µm, while the lateral resolutions are found to increase from 170 to 450 µm with increased depth, attributed to the divergence of the US propagation (Fig. 2c,d). The PA amplitude, affected by both the light intensity and overlap between optical beam and ultrasonic wave, was detected within a depth range from 1.4 to 4.6 mm (Fig. 2e), sufficient to image the entire arterial wall.

### Performance of sheath material

**Figure 3:**
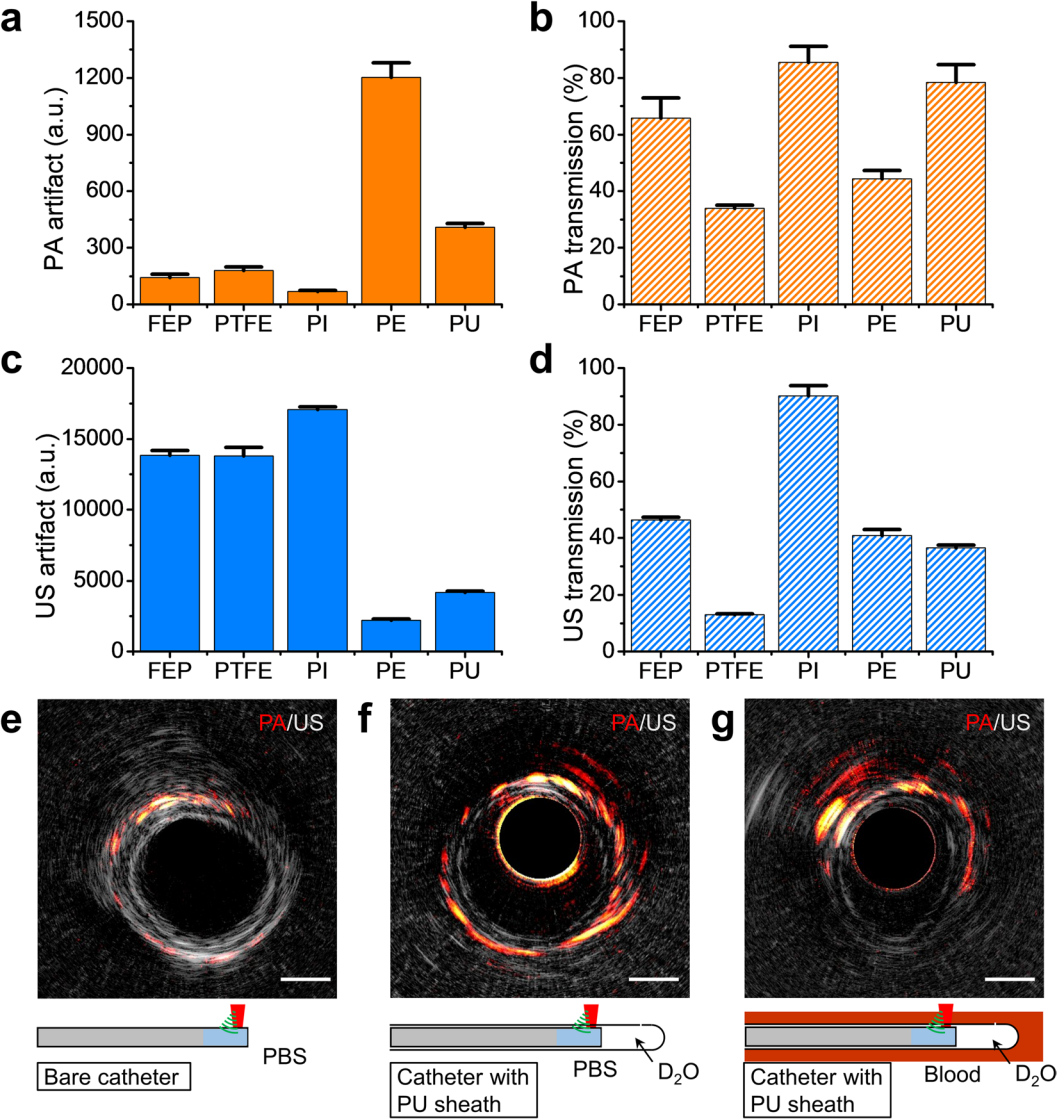
Performance of sheath material candidates. (**a**–**d**) Performance of five sheath material candidates by IVPA imaging of a heat-shrink tube to evaluate the signal transmission and artifact generation from the sheath. The value of artifact is regarded as the maximum signal from the sheath and the transmission is determined by comparing with bare catheter situation. The detailed imaging results can be found in Supplementary Fig. S2. (**e**–**g**) Comparative IVPA images of a human coronary artery imaged *ex vivo* in different environments: (**e**) bare catheter without a sheath and with luminal PBS, (**f**) catheter with D_2_O-filled PU sheath and luminal PBS, and (**g**) catheter with D_2_O-filled PU sheath and luminal blood. The scale bar is 1 mm for cross-sectional images. Error bars in (**a**–**d**) were resulted from five consecutive measurements of the target. FEP: fluorinated ethylene propylene, PTFE: polytetrafluoroethylene, PI: polyimide, PE: polyethylene, PU: polyurethane, PBS: phosphate-buffered saline.

A sheath for IVPA catheter is used to provide necessary protection to endothelia from damage by fast-rotating catheter as well as to the catheter from mechanical damage due to blood, thrombus, or the catheterization procedure. A functional IVPA sheath material should be optically and acoustically transparent, to reduce attenuation of PA and US signals to a minimum and induce minimal artifacts, which is still under discovery. To find a proper sheath material, we carefully selected a series of sheath material candidates based on their optical and acoustic properties (Supplementary Table S1) and tested their performance by imaging a heat-shrink tube with our quasi-collinear catheter. The imaging results are shown in Supplementary Fig. S2 with comparison with a bare catheter. Their performance in term of induced artifacts by the sheath and transmission over the sheath for PA and US signals was also summarized in Fig. 3a–d. Although fluorinated ethylene propylene, polytetrafluoroethylene, and polyimide induced minimal artifacts for PA images, their overwhelming US artifacts make them difficult to be selected as proper sheath materials (Supplementary Fig. S2b–d). Compared with polyethylene, polyurethane (PU) exhibits a smaller PA artifact, a larger PA transmission and comparable US behavior (Supplementary Fig. S2e,f and Fig. 3a–d), thus was selected as our material of choice for the sheath in imaging window section (Fig. 1b).

PU sheath with dimension adapted to the imaging catheter was further evaluated by *ex vivo* imaging of a human coronary artery in different environments (Fig. 3e–g). The catheter with a D_2_O-filled PU sheath demonstrated comparable or even stronger PA intensity and moderate US attenuation as compared to imaging with the bare catheter in phosphate buffered saline (PBS) (Fig. 3e,f). In other words, the optical loss across the sheath material was compensated by filling the sheath with D_2_O, which has a much smaller absorption coefficient than water at 1.7 µm^31^. Furthermore, IVPA imaging with PU sheath in the presence of luminal blood (Fig. 3g) demonstrated the capability of our imaging system for *in vivo* intravascular imaging without luminal blood flushing or occlusion, which is an important advantage over other optical imaging modalities such as optical coherence tomography in clinical applications. The following *in vivo* imaging experiments were based on the scheme described in Fig. 3g.

### *In vivo* IVPA imaging of rabbit aorta

**Figure 4:**
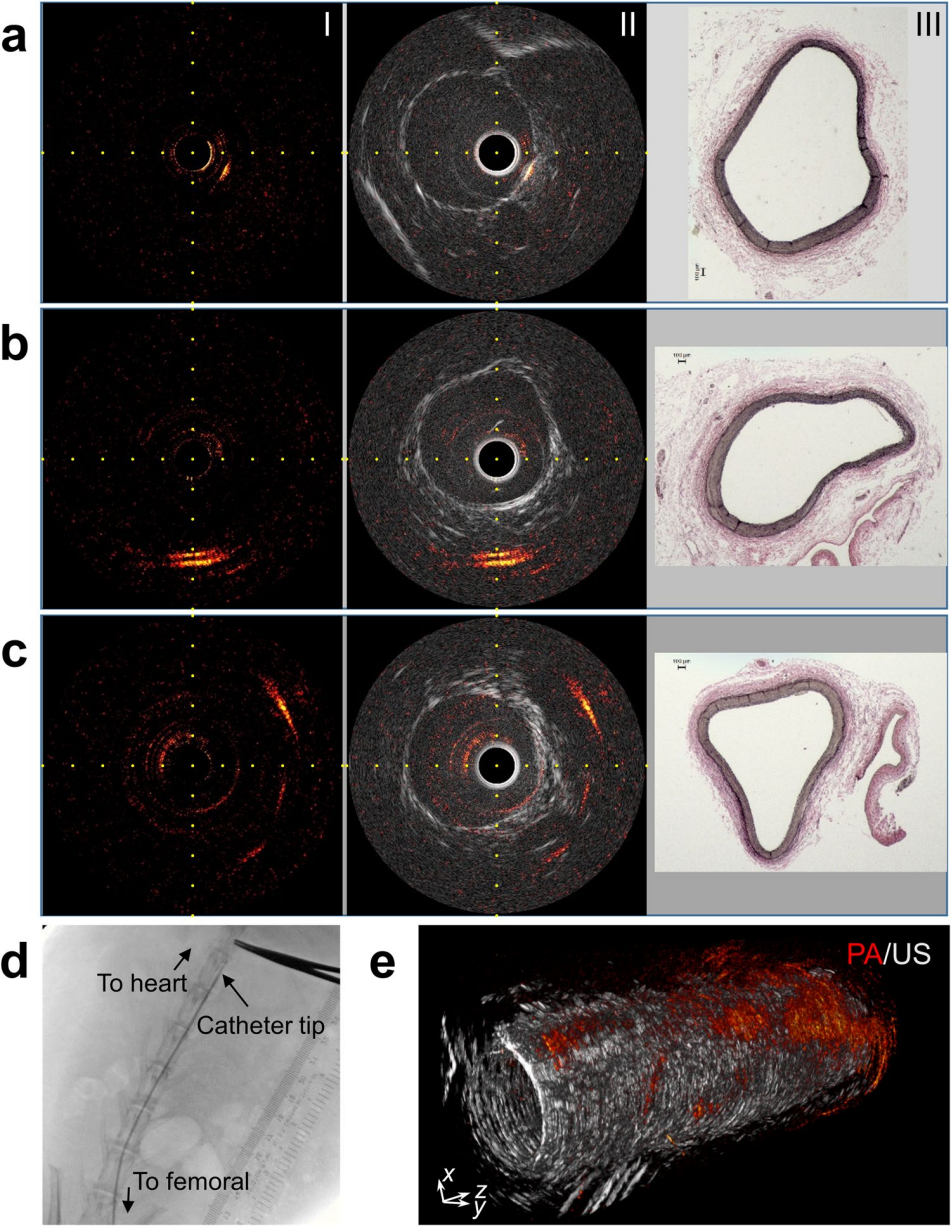
*In vivo* IVPA imaging of a rabbit aorta. (**a**–**c**) Intravascular PA and US images, and corresponding Verhoeff-van Gieson stained histopathology at different positions along aorta. Labels I-III correspond to PA, merged PA/US images, and histopathology, respectively. 1 mm grid scale was marked in PA/US images. (**d**) X-ray angiogram of IVPA catheter in the thoracic aorta, with forceps and ruler to locate the position of the catheter externally. (**e**) Reconstructed 3D merged PA/US image for a pullback segment of 20-mm length of the aorta. Images in this figure were collected at 4 fps and a pullback speed of 0.25 mm/s.

To validate the feasibility and performance of our imaging system *in vivo*, we imaged the thoracic aorta of three lean, male New Zealand White (NZW) rabbits. The catheter was placed through femoral artery under x-ray angiography (Fig. 4d). We recorded *in vivo* IVPA images of the aorta with 80-mm pullbacks at different rotational and pullback speeds, up to 16 fps and 1 mm/s (Supplementary Videos S1 and S2), respectively. Figure 4a–c shows representative cross-sectional PA (I), merged PA/US images (II), and histology results (III) at different positions corresponding to the distal, upper and proximal sections of thoracic aorta (Supplementary Fig. S3a). The PA images show the presence of lipid within the aorta wall (Fig. 4a) and perivascularly at depths greater than 4 mm (Fig. 4b,c). The US images provide important morphological information about the artery, such as luminal area and thickness of artery wall. Given the young age and lean diet of the NZW rabbits, we did not expect to see any vascular pathology and indeed the histology shows this. The abundance of perivascular adipose tissue agrees with the strong PA signals detected peripherally in the corresponding sections (Fig. 4b,c). Reconstructed 3-dimensional (3D) PA/US merged image with a 20-mm pullback length (Fig. 4e and Supplementary Fig. S4) illustrates the detection and presence of perivascular adipose tissue at the proximal end of the pullback, close to the femoral artery.

**Figure 5:**
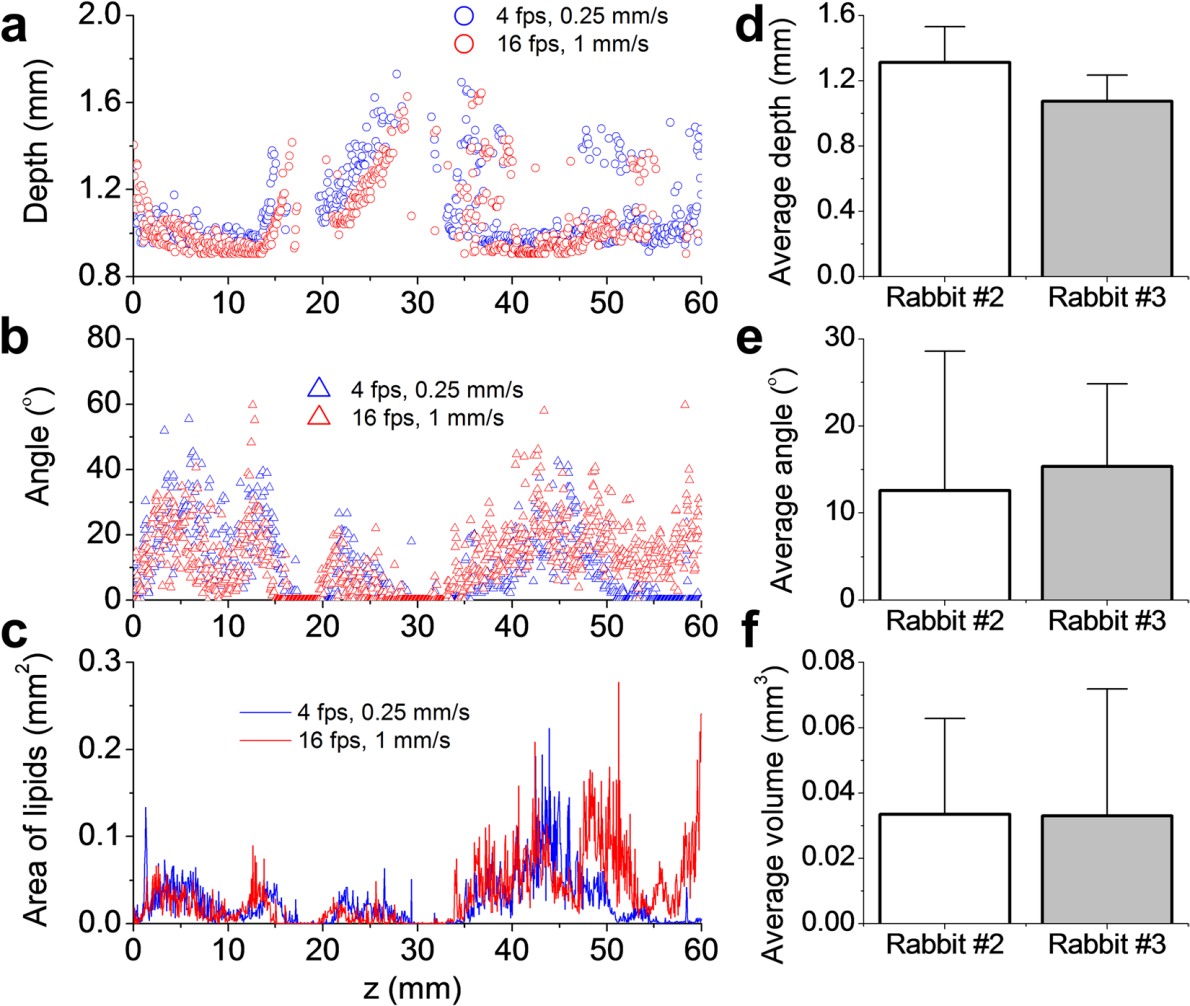
Quantification of lipid core in rabbit aortas *in vivo*. (**a**–**c**) Comparative result of quantitated lipid core depth (**a**), angle (**b**) and area of lipids (**c**) in a rabbit aorta (#3) at each frame along a pullback length of 60 mm with different rotational and pullback speeds (4 fps and 0.25 mm/s vs. 16 fps and 1 mm/s). Lipid core depth corresponds to the depth to catheter center where PA signal shows a maximum amplitude; angle of lipid core means the observation angle of the maximum lipid core from catheter center; area of lipids is obtained by counting all the lipids in and surrounding the arterial wall. (**d**–**f**) Average lipid core depth (**d**), angle (**e**), and volume of lipids in a 1 mm artery length (**f**) for two rabbit aortas (#2 and #3). The error bars are resulted from all the frames during entire pullbacks.

We further compared imaging performance by imaging the thoracic aorta of another rabbit in terms of lipid core depth, observation angle and lipid area (Supplementary Fig. S5) at different rotational and pullback speeds (4 fps and 0.25 mm/s vs. 16 fps and 1 mm/s). Similar results were observed (Fig. 5a-c and Supplementary Fig. S6), confirming the reproducibility of our imaging system and protocol. The averaged results for two rabbits along 60-mm pullbacks further confirmed the healthy aorta of the rabbits on lean diet (Fig. 5d–f).

### *Ex vivo* imaging of human coronary artery

**Figure 6:**
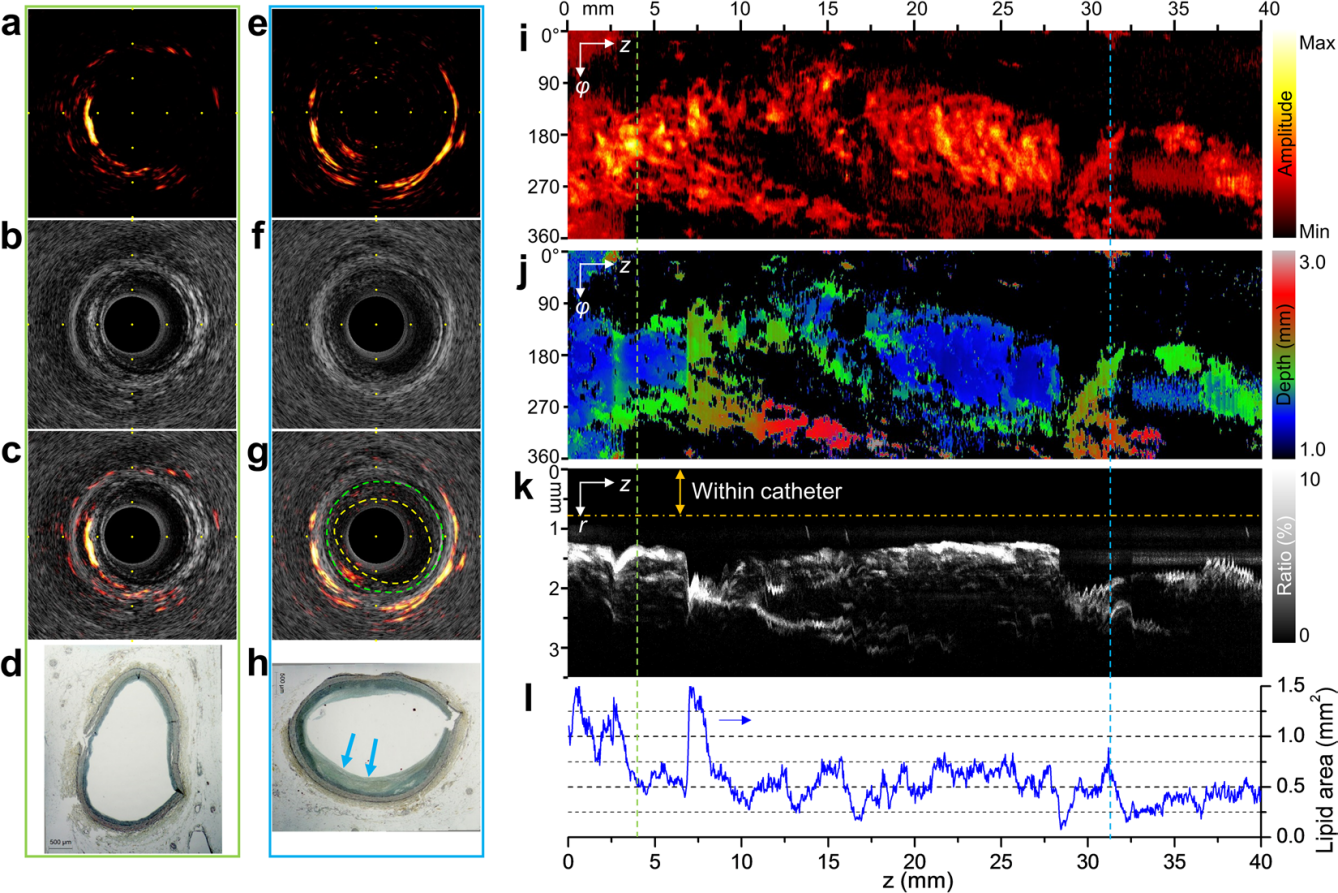
*Ex vivo* IVPA imaging of a human right coronary artery. (**a**,**e**) Representative cross-sectional PA images, (**b**,**f**) US images, (**c**,**g**) merged PA/US images and (**d**,**h**) their corresponding Movat’s pentachrome-stained histopathology sections. The frame locations along the artery are indicated by individual color lines in panel i–l. In panel g, the boundaries of lumen and external elastic membrane are outlined by yellow and green lines, respectively, to illustrate the intimal thickening observed on US image. Intimal thickening having lipid is shown by the arrows in panel h. (**i**) Maximum PA amplitude at each radial direction (*φ*) from 0 to 360° along pullback direction (*z*) from 0 to 40 mm, and (**j**) their corresponding depth from the center of the catheter. (**k**) Angular ratio of maximum lipid pool at individual depth along the artery. (**l**) Quantitated lipid area at each cross-section of the artery for the 40-mm pullback.

To validate the performance of our imaging system for the detection of true coronary pathology and future translational applications, we further implemented our imaging system on a human right coronary artery *ex vivo*. The IVPA catheter with sheath was advanced 40 mm into the distal artery and imaged at 16 fps and pullback speed of 0.5 mm/s with constant perfusion with PBS. Results are shown as cross-sectional PA (Fig. 6a,e), US (Fig. 6b,f) and merged PA/US (Fig. 6c,g) images. Corresponding histopathology result (Fig. 6d,h) with Movat’s pentachrome stain (see Methods) at representative locations was also displayed to confirm our observation. A short movie composed of merged PA/US images and their pullback view was provided in Supplementary Video S3. Strong PA signals were observed outside the vessel from perivascular adipose tissue, while obvious PA signal was detected from the thickened intima layer (7 o’clock) as well (Fig. 6e–g), which is very likely from lipid-rich plaque as highlighted by color outlines in Fig. 6g and confirmed by histology result in Fig. 6h (arrows). Additionally, *ex vivo* angiography with contrast shows a small lesion (indicated by arrowhead) approximately 10 mm from the introducer sheath (indicated by arrow) (Supplementary Video S4), corresponding to the thickened region in the histology section shown in Fig. 6h (arrows). The 2-dimensional lipid distribution and depth maps at the peaks of PA A-lines are shown for a 40-mm segment of the artery (Fig. 6i,j). Dense lipid distribution along the entire pullback was observed with a depth ranging from 1 mm to 3 mm. Angular ratio of the maximum lipid pools, i.e. the angle of view over 2π in percentage, at each individual depth was calculated frame by frame for the entire pullback (Fig. 6k and Supplementary Fig. S5), which further helps to quantify the lipid core size and depth in lipid-rich plaque identification. The total lipid area was quantitated for each cross-section along the artery (Supplementary Fig. S5) and presented with alignment to lipid distribution maps (Fig. 6i–k) to show the variation of lipid accumulation within and outside the vessel wall (Fig. 6l). The reconstructed 3D images in different views (Supplementary Fig. S7 and Supplementary Video S5) illustrate lipid distribution pattern in relation to the artery morphology.

## Discussion

IVPA imaging brings forth novel capabilities for the detection of lipid-rich atherosclerotic plaques and perivascular adipose tissue without displacement or occlusion of blood flow. To address the challenges of *in vivo* implementation, we developed a high-sensitivity quasi-collinear IVPA catheter and performed a comprehensive study on the selection of a sheath material. These advances enabled *in vivo* demonstration of IVPA imaging of rabbit aortas under clinically relevant conditions, i.e. imaging with a catheter sheath in presence of luminal blood and real-time display at 16 fps, which presents a key step towards clinical translation. *Ex vivo* evaluation on human artery exhibited the significance of our imaging system in the localization and quantification of lipid deposition across the entire arterial wall, including perivascular adipose tissue. It is increasingly accepted that atherosclerotic lesions primarily develop in arteries with perivascular adipose^32^ and surgical removal of the adipose encasing the arteries attenuates atherogenesis^33^.

Several further improvements are necessary to achieve clinical application. First, the diameter of our current imaging catheter and sheath needs to be further reduced from 1.6 mm to ~1.0 mm for safe coronary artery access. This can be achieved with a thinner optical fiber and rod mirror, smaller diameter torque coil, better integration of catheter components, and thinner catheter sheath. Second, the sheath material can be further optimized from more polymer candidates to further improve the imaging quality by reducing the transmission losses and avoiding unnecessary artifacts from the sheath. Third, a broadband transducer covering the low-frequency PA signal, typically in several MHz range^34^, while maintaining US resolution needs to be developed for better imaging quality. In addition, all materials used for catheter fabrication should adhere to regulatory control for biosafety. The clinical goals of IVPA imaging will be within reach by implementing these technical improvements.

As intraplaque hemorrhage is deemed as a common phenomenon in advanced coronary atherosclerotic lesions and could be an important indicator for plaque rupture^35^, integrating intraplaque blood detection in the future IVPA system design could be beneficial. This can be implemented by involving another laser wavelength with strong absorption for blood, for example 532 nm. However, such development may increase the complexity of the system and slow down the imaging speed, as well as raise the necessity of temporal luminal blood clearance during intravascular imaging due to strong optical attenuation.

The broad goal of IVPA imaging is to provide a foundation for building a multimodal platform for imaging lipid-laden, vulnerable plaque^36^ due to its unique capabilities of chemically-specific and depth-resolved detection of lipids. Significant value of IVPA imaging may be seen in several areas: 1) Characterization of the natural history and progression of vulnerable plaque; 2) Identification of solitary vulnerable plaque to determine the efficacy of treatment interventions; 3) Determination of the efficacy of preventative therapies (e.g. statins) to reduce lipid-core size^37^. Multimodal IVPA-IVUS imaging could open opportunities beyond the reach of other intravascular imaging tools^36,38^.

## Methods

### A portable high-speed IVPA tomography system

The high-speed IVPA tomography system developed by our group provided dual-modality intravascular photoacoustic and ultrasound imaging at speed up to 16 fps with real-time display (Supplementary Fig. S1)^22^. A Nd:YAG pumped OPO (Nanjing Institute of Advanced Laser Technology) emitting ~10-ns pulse with 2-kHz repetition rate at a wavelength of 1730 nm served as the excitation laser source. The laser beam was coupled to the imaging catheter via a multimode fiber and then directed to the arterial wall for lipid-specific excitation. A customer-designed hybrid optical and electrical rotary joint allowed for efficient optical coupling and radiofrequency signal transmission at fast rotation. A self-designed and -assembled quasi-collinear IVPA catheter with an outer sheath was used for intravascular PA/US imaging (Fig. 1b). For safety, the output pulse energy from the catheter tip was controlled around 100 µJ, corresponding to a laser fluence of 50 mJ/cm^2^ and below the ANSI laser safety standard of 1 J/cm^2^ at 1730 nm. Delayed (5 µs in this work) ultrasound pulses triggered by a pulse generator (Model 9512, Quantum Composers, Inc.) were sent/received by a pulser/receiver (5073PR, Olympus, Inc.) to provide co-registered ultrasound image of the artery. A computer with 500-MS/s 12-bit DAQ card (ATS9350, AlazarTech, Inc.) was used for control, processing, real-time display, and data collection. The entire system was installed in a portable cart for easy movement (Supplementary Fig. S1b).

### Quasi-collinear IVPA catheter design

A quasi-collinear IVPA catheter design was developed for high sensitivity *in vivo* application (Figs 1b and 2a). A multimode fiber (FG365LEC, Thorlabs) was used for high-power laser pulse delivery. A fiber-end mirror polished to 45° and coated with gold was used for optical direction to the artery wall. An US transducer (0.5 × 0.6 × 0.2 mm^3^, 42 MHz, 50% bandwidth) (AT23730, Blatek Industries, Inc.) served for PA detection and US pulsing/receiving. The transducer was positioned next to the rod mirror and tilted by 10° forward to maximize the overlap between US and optical waves to realize a quasi-collinear PA detection, and to reduce the multiple US reflection from the protective sheath. The overlap depth is estimated from 0.6 to >6 mm by geometrical calculation considering the dimension of components and reasonable divergence angles of 6° for optical beam and 3° for US wave. The components were positioned in a 3D printed plastic housing (Proto Labs) and further protected by a stainless steel tube. The catheter rotation was transferred to the tip via a torque coil. A custom-designed sheath was used to protect the entire sheath for *in vivo* application.

### Selection of sheath material

In order to find a proper sheath material, we selected five different polymers as candidate based on their optical and acoustic properties, i.e. low optical absorption at 1.7 μm and matched acoustic impedance with aqueous medium (Supplementary Table S1). To test their PA and US behavior, the polymers were fabricated into tubes with proper dimension to fit the IVPA catheter, and a heat-shrink tube was imaged with/without these sheath material (Supplementary Fig. S2). PA/US artifact generated from and transmission over the sheath were analyzed to provide criteria for sheath material selection.

### Lipid quantification

Cross-sectional PA images was reconstructed according to Supplementary Fig. S5. The maximum PA intensity along the radial direction and its corresponding depth from the catheter center were calculated for each frame (Supplementary Fig. S5b,c) to generate two-dimensional maps of lipid presence and depth (Supplementary Fig. S5d,e), which provides an overview of depth-resolved lipid distribution. A binary lipid index image (i.e. 0 for background and 1 for lipid) was generated by applying a predefined threshold (4 times of background noise in this work) to the PA images (Supplementary Fig. S5f). The value of the threshold was selected from a series of integral times of the background noise and determined by optimal match between resulted lipid index map and PA image. The angular ratio of biggest lipid pool at each depth, i.e. angle of field of view over 2π, was generated for every frame (Supplementary Fig. S5g,h) and plotted for the entire pullback length (Supplementary Fig. S5i) to give complementary information about the lipid-core size and depth. The lipid area in each frame was calculated based on the binary lipid index image and plotted against the pullback length to visualize the total lipid deposition longitudinally (Supplementary Fig. S5j).

### Procedure for *in vivo* IVPA imaging of rabbit aortas

This protocol was performed according to the *Animal Studies for Cardiovascular and Intestinal Imaging* and approved by the Purdue Animal Care and Use Committee. Three male NZW rabbits (Charles River Laboratories), aged eight months old and fed with a normal chow diet, were used for *in vivo* IVPA imaging. Before imaging procedure, the rabbit was anesthetized with a proper dose of ketamine (35 mg/kg) and xylazine (5–10 mg/kg) through ear vein injection and maintained on 1–5% isoflurane mixed with 100% O_2_ via endotracheal intubation during the entire imaging process. A cutdown procedure was used to identify the left femoral artery for intravascular access. A 6 Fr introducer sheath was inserted in the femoral artery, through which the IVPA catheter was advanced to the thoracic aorta (Supplementary Fig. S3a,b), guided by x-ray angiography. The catheter sheath was flushed with D_2_O to reduce optical loss and remove laser heating during IVPA imaging. Different rotational and pullback speed combinations (4 fps and 0.25 mm/s, 16 fps and 1 mm/s) were used to confirm the reproducibility of our imaging system. A total length of 80 mm was recorded for each pullback. Following imaging, the rabbit was euthanized by using intravenous euthanasia solution (390 mg/ml) and the aorta was harvested for histology (Supplementary Fig. S3c).

### Human coronary artery preparation

The experiments of human tissue samples were approved by Human Research Protection Program of Purdue University and performed in accordance with the approved guidelines. The informed consent was obtained from all subjects. A fresh, human heart was harvested from a 44-year-old female undergoing transplant surgery within 24 hours. Immediately, the coronary arteries were excised and cannulated with a 6 Fr introducer sheath, sutured in place (Supplementary Fig. S7a). The artery was then pinned flat into a container and submerged in 1 × PBS. The IVPA catheter with sheath was advanced distally, approximately 40 mm past the introducer sheath. During imaging, the artery was perfused with 1 × PBS at room-temperature and catheter was flushed with D_2_O. Pullback was recorded at 16 fps and 0.5 mm/s for a total length of 40 mm.

### Histology approaches

All arteries were pressure fixed in 10% w/v formalin at approximately 25 mL/min for 30 minutes to maintain lumen as close to *in vivo* morphology as possible. The arteries were then grossly sectioned in 3–4 mm segments and paraffin embedded, sectioned, and stained for Verhoeff-van Gieson and Russel-Movat’s pentachrome.

## Electronic supplementary material

Supplementary Information

Video S1

Video S2

Video S3

Video S4

Video S5
