# Supplementary material for: Fast assessment of lipid content in arteries in vivo by intravascular photoacoustic tomography
Source: Sci Rep. 2018 Feb 5;8:2400. doi: 10.1038/s41598-018-20881-5 (PMC5799328; doi:10.1038/s41598-018-20881-5)
Supplement: Supplementary file 1 — Supplementary Information [file 41598_2018_20881_MOESM1_ESM.docx]

**Supplementary Information**

**Fast assessment of lipid content in arteries *in vivo* by intravascular photoacoustic tomography**

Yingchun Cao, et al.

**Supplementary Figures:**


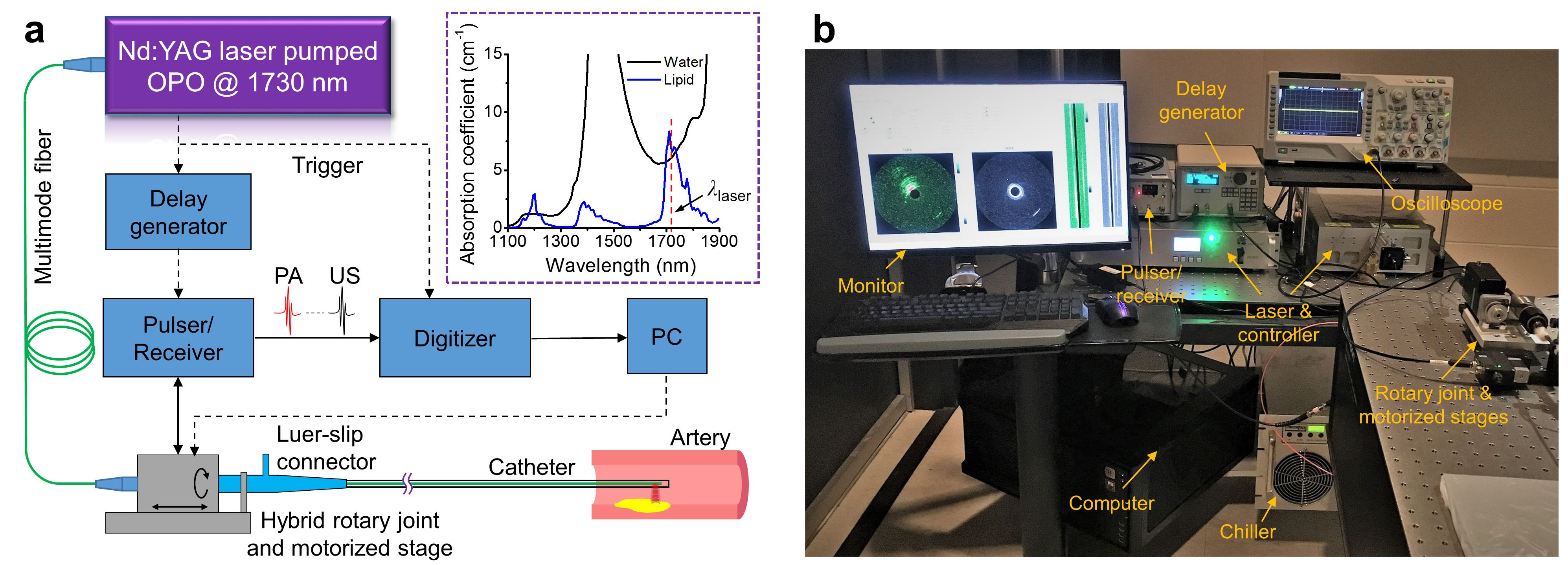


**Supplementary Fig. S1** Schematic and photograph of the intravascular photoacoustic (IVPA) imaging system. (**a**) Schematic setup of the IVPA imaging system. The inset purple box shows the absorption spectra of water and lipid in near infrared spectral range. A lipid-specific imaging wavelength of 1730 nm was used due to the strong absorption peak of the first vibrational overtone transition of C-H bond, which is abundant in lipids, and the absorption valley of water in this spectral range[^1^](#_ENREF_1)^,^[^2^](#_ENREF_2). Although the difference in absorption coefficients of lipid and water at 1730 nm is not very distinct, the generated photoacoustic signal from lipid versus water is substantial due to the greater Gruneisen parameter of lipid[^3^](#_ENREF_3), allowing for ample contrast. (**b**)Photograph of the portable IVPA system. OPO: optical parametric oscillator, PA: photoacoustic, PC: personal computer, US: ultrasound.


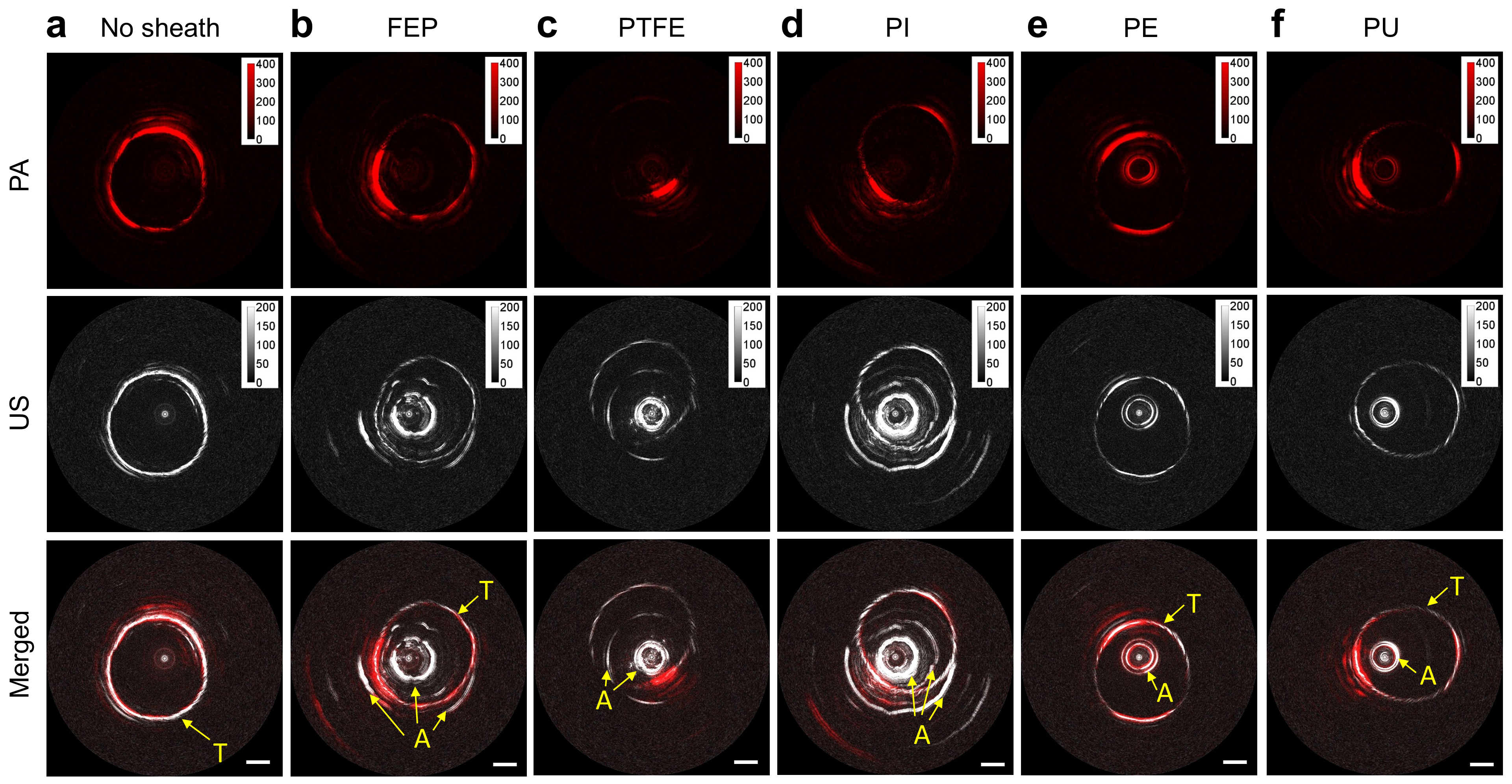


**Supplementary Fig. S2** Evaluation of different polymer tubing serving as a sheath material for the IVPA catheter. Heat-shrink tubing with a diameter of ~4 mm serves as the imaging target due to its strong photoacoustic signals attributed to the compositions of polyolefin and black carbon. The catheter rotates inside the sheath to obtain photoacoustic/ultrasound (PA/US) images of the target. (**a**) The bare catheter without a sheath serves as a control. (**b-f**) Imaging results for sheath material candidates. Both imaging target (T) and induced artifacts (A) by the sheath are indicated in the merged PA/US images. FEP: fluorinated ethylene propylene, PTFE: polytetrafluoroethylene, PI: polyimide, PE: polyethylene, PU: polyurethane. The scale bar is 1 mm. There are notable ultrasound artifacts for FEP, PTFE, and PI (**b-d**). Notable photoacoustic artifact is observed for PE (**e**).

**
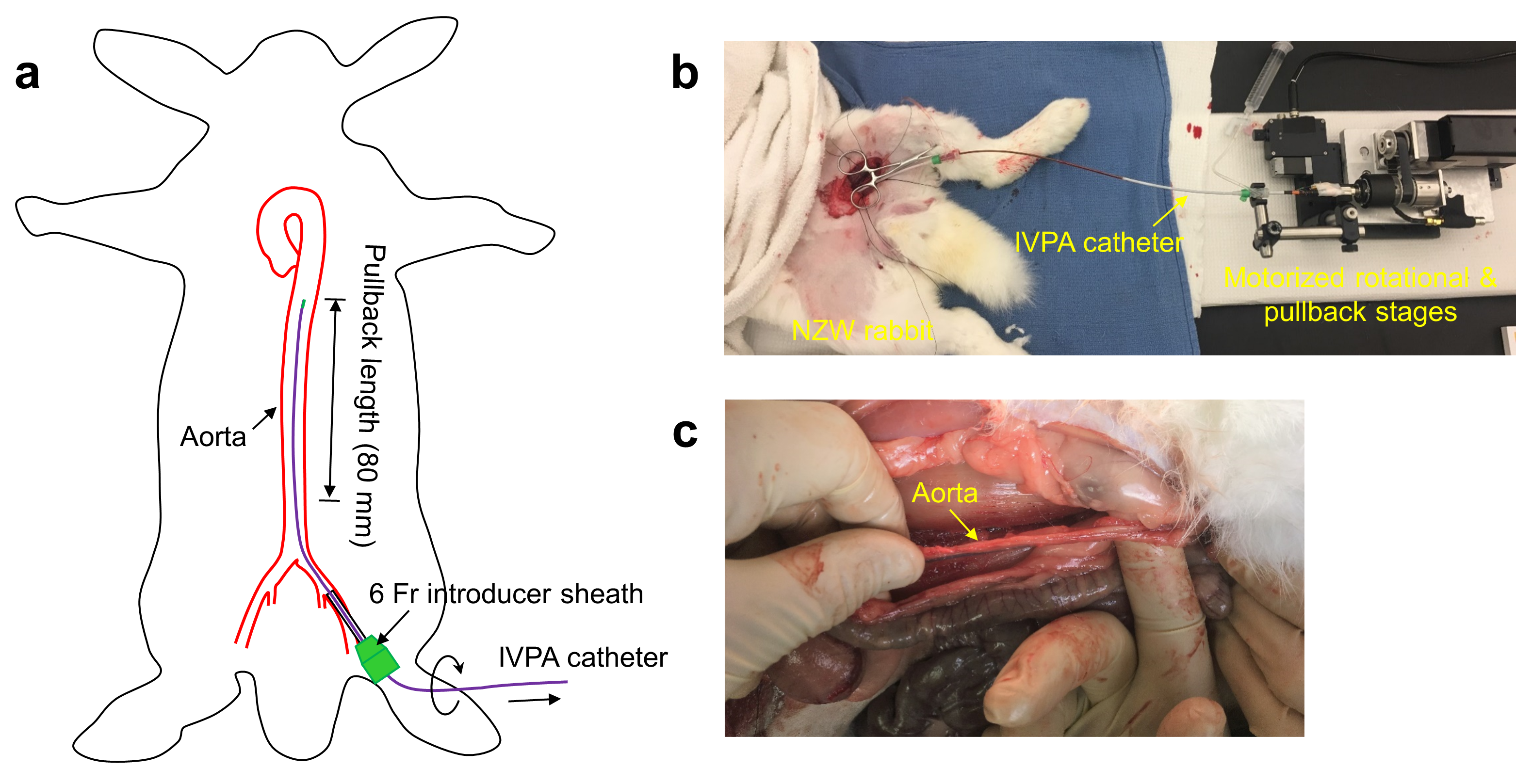
**

**Supplementary Fig. S3** Surgical procedure for *in vivo* IVPA imaging of a New Zealand White (NZW) rabbit. (**a**) Plan for *in vivo* IVPA imaging of rabbit aorta with a pullback length of 80 mm. (**b**) 6 Fr introducer sheath was used to access the left femoral artery for catheterization. (**c**) Aorta was excised for histology.


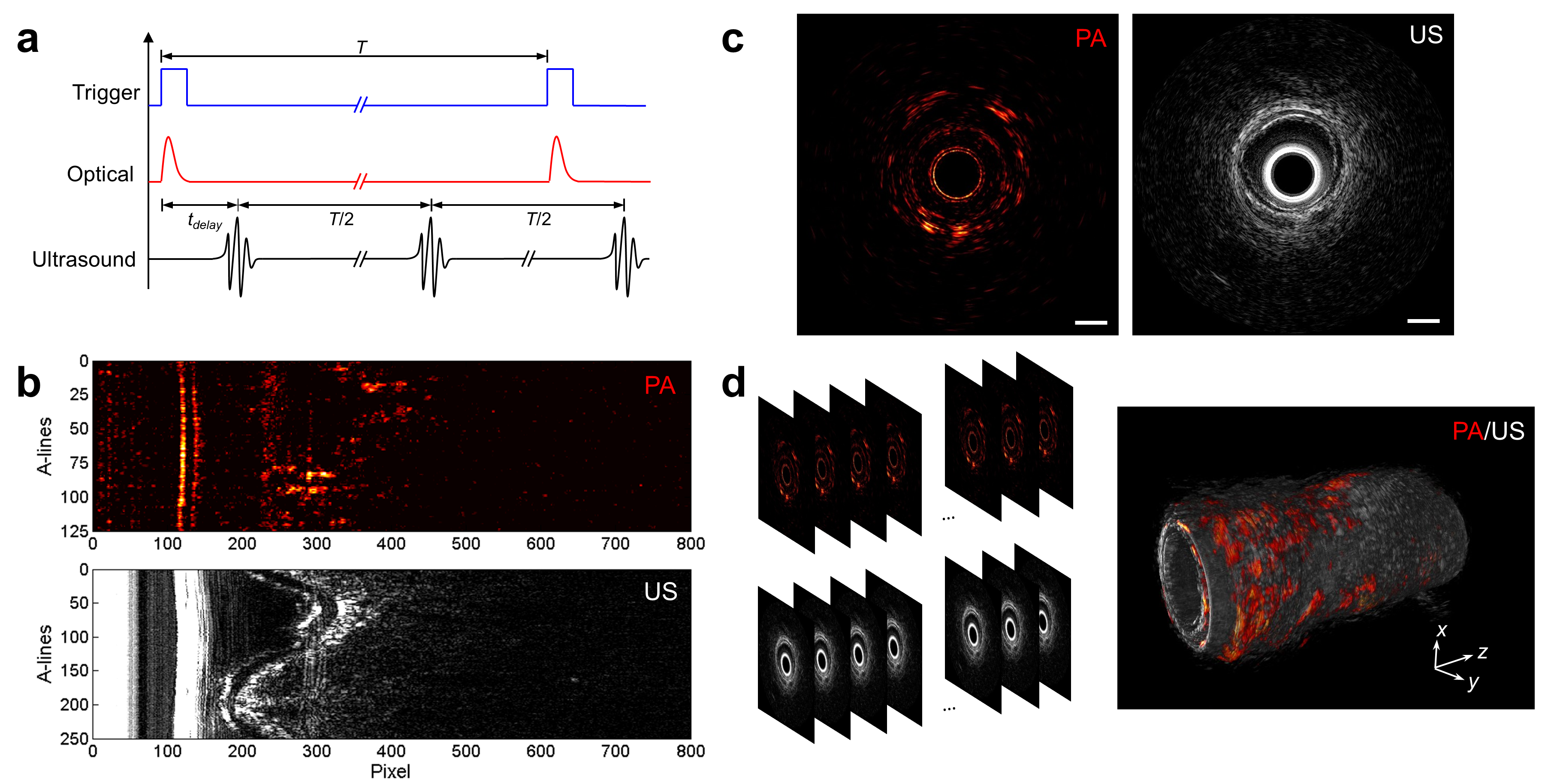


**Supplementary Fig. S4** Procedure for IVPA data acquisition and processing. (**a**) Trigger signal was generated by the excitation laser source and synchronized with optical pulses; ultrasound pulses with double frequency and 5-µs delay to optical pulses were sent by ultrasound pulser/receiver to generate co-registered and definition-improved IVUS image for high-speed real-time imaging. (**b**) A-lines for both PA and US channels after bandpass filtering, Hilbert transform, and noise removal. (**c**) Cartesian coordinate expression of PA and US images with designated pixel density of 80 pixel/mm and scale bar of 1 mm. (**d**) 3-dimensional (3D) PA and US images reconstructed from their cross-sectional image stacks with merged display.

**
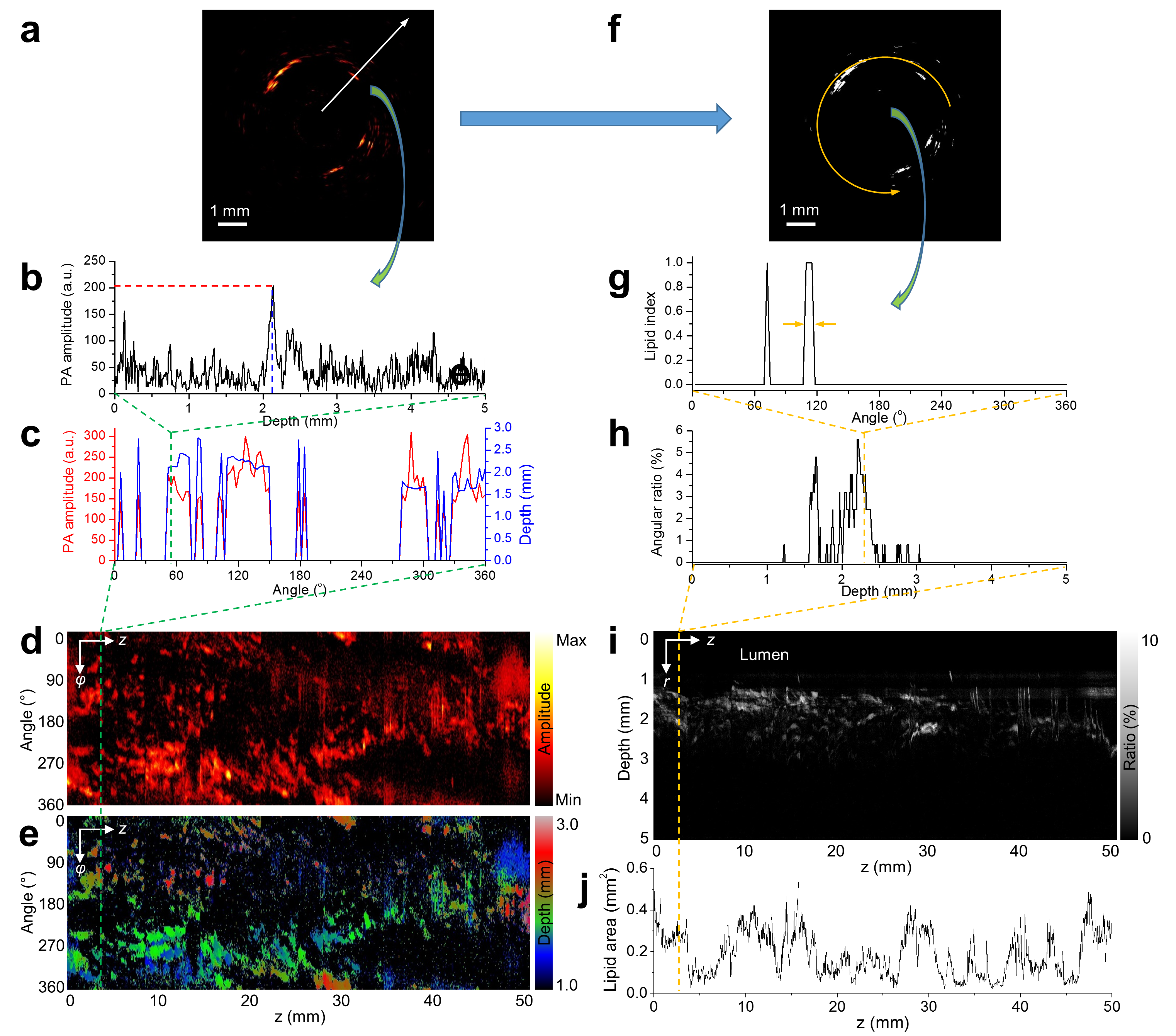
**

**Supplementary Fig. S5** Procedure for lipid quantification and localization. (**a**) Cross-sectional photoacoustic image was reconstructed from the raw data. (**b, c**) Peak amplitude of photoacoustic signal along the radial direction was detected and the corresponding depth was recorded for each frame. (**d**, **e**) Peak amplitude of photoacoustic signal and depth were expressed as 2-dimensional images for the entire pullback to indicate lipid distribution and depth. (**f**) A proper threshold (4 times of noise level in this work) was applied to photoacoustic image to generate a cross-sectional binary lipid map (i.e. 0 for background and 1 denotes lipid presence). (**g**) Lipid presence along angular direction at a specific depth was plotted to the show the angle of view for lipid pools. (**h**) The angular ratio of the largest lipid pool, i.e. angle of view over 2π in percentage, was generated for each depth. (**i**) A map of angular ratio of largest lipid pool was produced along the longitudinal direction of the artery to provide a complementary information about the lipid pool size and distribution depth. (**j**) The total lipid area for each cross-section was quantitated from (**f**) for the entire artery.

**
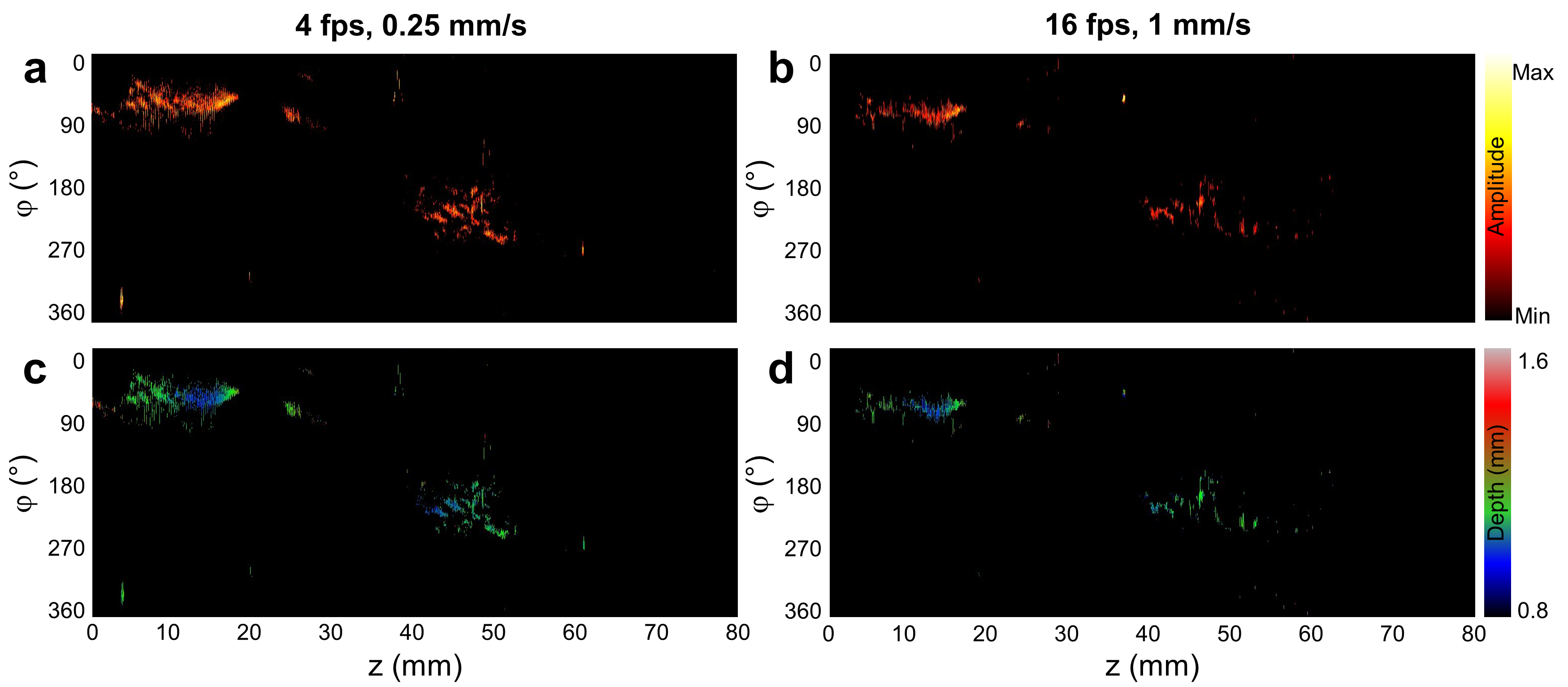
**

**Supplementary Fig. S6** A comparison between two pullbacks of *in vivo* IVPA imaging of a rabbit aorta with different rotational and pullback speeds. (**a**, **b**) Maps of peak photoacoustic amplitude and (**c**, **d**) corresponding depth. **a**, **c** correspond to rotational speed of 4 fps and pullback speed of 0.25 mm/s, while **b**, **d** are for 16 fps and 1 mm/s.

**
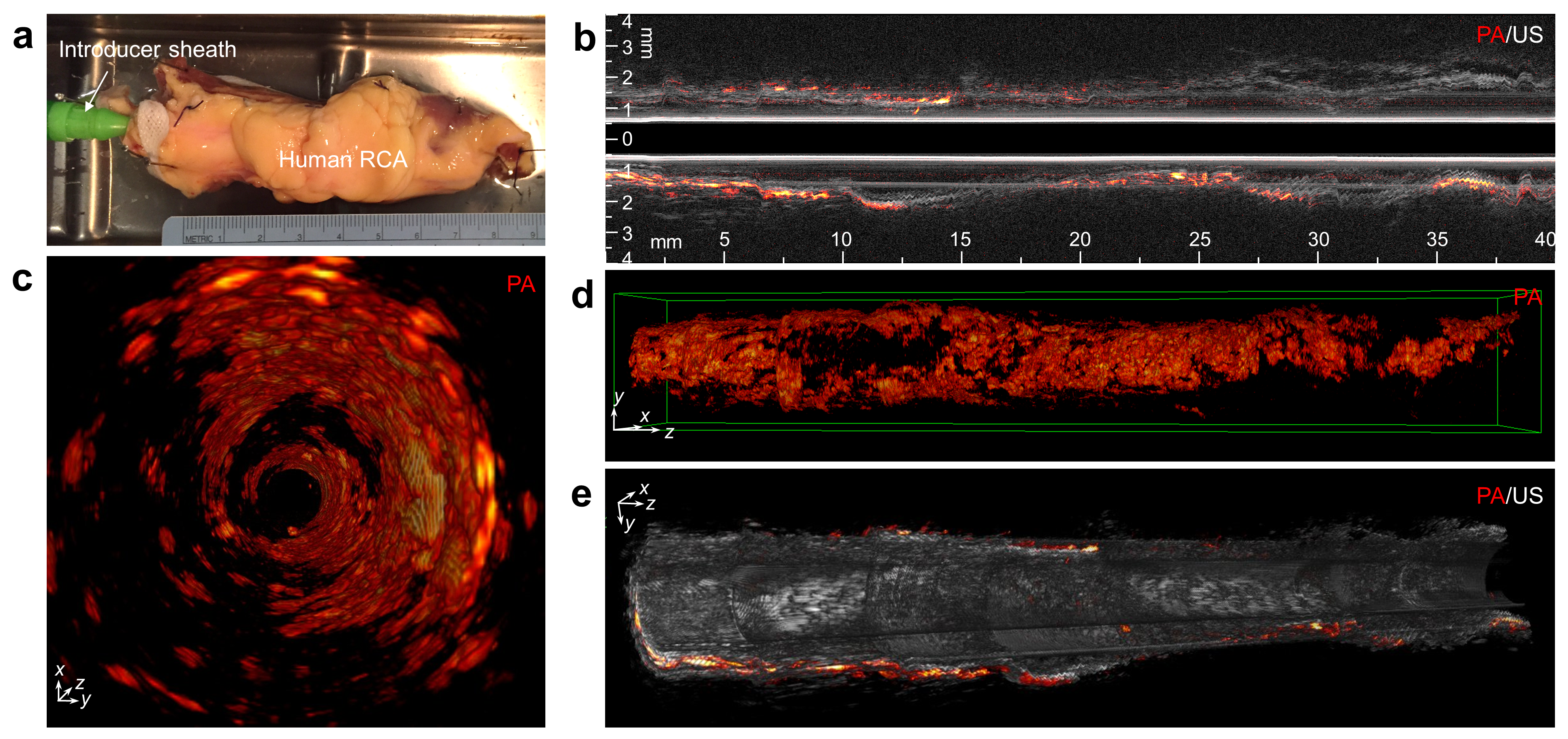
**

**Supplementary Fig. S7** Human right coronary artery (RCA) and imaged results. (**a**) Photograph of gross human RCA, cannulated at the ostium with an introducer sheath for IVPA catheter advancement. (**b**) Imaged PA/US result of the artery with a pullback length of 40 mm in a cutaway view along pullback direction. (**c**) Imaged lipid distribution in a fly-through view from inside the artery. (**d**) 3D reconstructed lipid distribution in the artery in side view. (**e**) Reconstructed 3D PA/US merged images of the RCA with hemi-section display.

**Supplementary Table:**

**Supplementary Table S1** Optical and acoustic properties of sheath material candidates and liquid media[^4-6^](#_ENREF_4).

| Material |  | Optical properties | | |  | Acoustic properties | | | |  | Chemical Structure |
| --- | --- | --- | --- | --- | --- | --- | --- | --- | --- | --- | --- |
|  |  | *n* | *µ_a_* (cm^-1^) | *µ_s_* (cm^-1^) |  | *ρ* (kg/m^3^) | *c_s_* (m/s) | *Z* (MPa s/m) | *α_s_* (dB/cm) |  |  |
| Water |  | 1.31 | 7.41 | 0.07 |  | 1000 | 1450 | 1.45 | 3.472 |  | 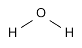 |
| Heavy water |  | 1.31 | 0.12 | 0.07 |  | 1000 | 1450 | 1.45 | 3.472 |  | 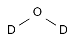 |
| PI |  | 1.7 | 2^*^ | - |  | 1420 | 2246 | 3.19 | - |  | 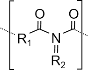 |
| PE (LD) |  | 1.5 | 10^*^ | - |  | 920 | 2080 | 1.91 | - |  | 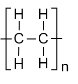 |
| PU |  | 1.5 | 4^*^ | - |  | 1200 | 1900 | 2.28 | - |  | 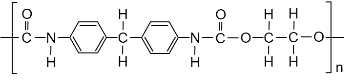 |
| FEP |  | 1.34 | 0.1^*^ | - |  | 2150 | 1330 | 2.86 | - |  | 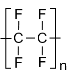 |
| PTFE |  | 1.34 | 0.1^*^ | - |  | 2160 | 1400 | 3.02 | - |  | 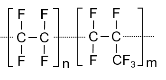 |

PI, Polyimide; PE, Polyethylene; LD, low density; PU, Polyurethane; FEP, Fluorinated ethylene propylene; PTFE, Polytetrafluoroethylene. *n*, refractive index; *µ_a_*, absorption coefficient; *µ_s_*, scattering coefficient; *ρ*, density; *c_s_*, speed of sound; *Z*, acoustic impedance; *α_s_*, acoustic loss. The optical properties correspond to optical wavelength of 1.7 µm and acoustic loss is for a frequency of 40 MHz. ^*^: estimated from their chemical structure and photoacoustic signals.

**Supplementary Videos:**

**Supplementary Video S1** IVPA imaging of a rabbit aorta *in vivo*. The catheter was rotated at 16 revolutions per second and pulled back at 0.5 mm/s.

**Supplementary Video S2** Real-time display of *in vivo* IVPA imaging of a rabbit aorta at 16 fps. Photoacoustic signals from perivascular adipose tissue and periodical artery movement due to heart beat can be visualized.

**Supplementary Video S3** Processed result for *ex vivo* imaging of a human RCA at 16 fps. Merged cross-sectional PA/US images and corresponding pullback views are displayed.

**Supplementary Video S4** Angiogram of a section of human RCA with contrast. The cannulated introducer sheath (arrow) and a small lesion ~10 mm on the right side of it (arrowhead) are shown.

**Supplementary Video S5** Reconstructed 3D images of a human RCA with a 40-mm pullback. Lipid content in different views and merged photoacoustic (red hot) and ultrasound (gray) images are displayed.

**Supplementary References:**

1. Wang, H. W. *et al.* Label-free bond-selective imaging by listening to vibrationally excited molecules. *Phys. Rev. Lett.* **106**, 238106 (2011).

2. Wang, P., Rajian, J. R. & Cheng, J. X. Spectroscopic imaging of deep tissue through photoacoustic detection of molecular vibration. *J. Phys. Chem. Lett.* **4**, 2177-2185 (2013).

3. Hui, J. *et al.* Bond-selective photoacoustic imaging by converting molecular vibration into acoustic waves. *Photoacoustics* **4**, 11-21 (2016).

4. <http://boedeker.com/mguide.htm>.

5. <http://www.shenitech.com/support/support_soundspeed.htm>.

6. <http://www.signal-processing.com/table.php>.
